# Contribution of TIP30 to chemoresistance in laryngeal carcinoma

**DOI:** 10.1038/cddis.2014.424

**Published:** 2014-10-16

**Authors:** M Zhu, F Yin, L Yang, S Chen, R Chen, X Zhou, W Jing, X Fan, R Jia, H Wang, H Zheng, J Zhao, Y Guo

**Affiliations:** 1 International Joint Cancer Research Institute, The Second Military Medical University, 800 Xiangyin Road, Shanghai 200433, People's Republic of China; 2 Changhai Hospital, The Second Military Medical University, 800 Xiangyin Road, Shanghai 200433, People's Republic of China; 3 PLA General Hospital Cancer Center, PLA Postgraduate School of Medicine, 28 Fuxing Road, Beijing, People's Republic of China

## Abstract

Laryngeal squamous cell carcinoma (LSCC) is one of the most common carcinomas of the head and neck. Despite advances in diagnosis and treatment, the survival of patients with LSCC has not improved in the past two decades. TIP30, a newly identified tumour suppressor, appears to be involved in multiple processes during tumour development. Here, we investigated the involvement of TIP30 in chemoresistance of LSCC *in vitro* and *in vivo*. We showed that TIP30 expression decreased significantly in drug-selected cells (DSCs) of laryngeal carcinoma. Suppressing TIP30 enhanced resistance capability to multiple chemotherapy drugs, cell proliferation and self-renewal in Hep2 cells. Additionally, decreased self-renewal capacity and chemotherapeutic resistance were observed in DSCs overexpressing TIP30. Furthermore, TIP30 negatively regulated tumourigenesis and chemoresistance in LSCC cells subcutaneously transplanted into nude mice. Moreover, decreased TIP30 expression contributed to chemoresistance, self-renewal and proliferation of LSCC cells via nuclearlisation of *β*-catenin, a cell–cell adhesion and stem cell renewal regulator. Consistently, Kaplan–Meier and Cox proportional hazards regression modelling analyses showed that decreased TIP30 expression independently predicted poor survival in patients with LSCC. Taken together, our results reveal that TIP30 has a crucial role in chemoresistance of LSCC through the AKT/glycogen synthase kinase-3*β/β*-catenin signalling pathway and may be a promising candidate for improving LSCC chemotherapy.

Laryngeal squamous cell carcinoma (LSCC) is one of the most common carcinomas of the head and neck, and surgery, radiotherapy and chemotherapy are the main treatments. The therapeutic effect is moderate in early-stage LSCC, but poor in advanced stages, despite improvements in management and treatment of the disease.^1,2^ Recurrence after surgery and resistance to chemotherapy are responsible for the poor prognosis of LSCC. Therefore, better understanding the molecular mechanisms of LSCC progression and identifying more effective targets to improve therapeutic efficacy for this disease are crucial.

A major obstacle in cancer treatment is the development of multidrug resistance. Drug-selected cells (DSCs) have been established to further explore the mechanism of multi-chemoresistance. DSCs, which are enriched cells resistant to chemotherapeutic agents,^3, 4, 5^ are relatively quiescent and are an efficient model for multidrug resistance research, as they preclude additional effects of chemotherapy-sensitive tumour cells. The properties of DSCs include not only multidrug resistance^6^ but also a stem-like phenotype,^7^ characterised by enhanced tumour initiation,^8,9^ metastasis^10^ and recurrence.^11, 12, 13^

TIP30, also called CC3 or HTATIP2, is a tumour suppressor that was originally identified by a differential display analysis of mRNA from the highly metastatic human variant small-cell lung cancer (SCLC) *versus* a less metastatic SCLC cell line.^14^ TIP30 is positively expressed in many normal human tissues but decreased expression is observed in some tumour tissues such as melanoma, breast cancer, neuroblastoma, glioblastoma, colon cancer and hepatocellular carcinoma (HCC).^14, 15, 16, 17^ TIP30 has tumour suppressor activity by inhibiting tumour growth,^18^ invasion^14^ and angiogenesis,^17^ and inducing apoptosis.^19^ TIP30 mutants not only abrogate tumour suppressor potential but also gain oncogenic activity.^20^ Moreover, *Tip30*^–^ mice exhibit a higher incidence of spontaneous tumour formation than wild-type mice,^15^ indicating a critical tumour-initiating function by TIP30. Forced expression of TIP30 sensitises HCC cells to 5-flurouracil (5-FU) and significantly inhibits growth of HCC xenografts in mice when combined with 5-FU.^18^ Our previous studies revealed that decreased TIP30 expression induces the epithelial–mesenchymal transition (EMT), a process related to tumour stemness, including self-renewal and chemotherapeutic resistance^21^ in pancreatic cancer cells.^22^ Additionally, sorafenib, an angiogenesis inhibitor, promotes invasiveness and metastasis of HCC in mice by downregulating TIP30 and inducing the EMT.^23^

The role of TIP30 as an important tumour suppressor in the development and progression of LSCC has not been characterised. In this study, we extensively investigated the contribution of TIP30 to drug resistance in LSCC. We demonstrated that TIP30 was greatly diminished in drug-selected LSCC cells. Forced expression of TIP30 greatly enhanced chemotherapeutic sensitivity of LSCC cells and prolonged survival of LSCC-xenografted mice. More importantly, our results suggest that decreased TIP30 expression enhanced chemotherapeutic resistance and self-renewal abilities of LSCC cells by modulating nuclearlisation of *β*-catenin. We further investigated the correlation between TIP30 expression and clinicopathologic characteristics and revealed its prognostic potential in patients with LSCC.

## Results

### TIP30 expression decreases in drug-selected LSCC cells

BALB/c nude mice were inoculated with Hep2 cells (LSCC cell line) and received an intraperitoneal injection of cisplatin (2 *μ*g/g) or phosphate-buffered saline (PBS) weekly for 5 weeks (Supplementary Figure S1). The mice were killed 6 weeks after inoculation; tumour cells were obtained by fine mincing and were digested with collagenase. The tumour cells from cisplatin-treated mice were called DSCs.

**Figure 1:**
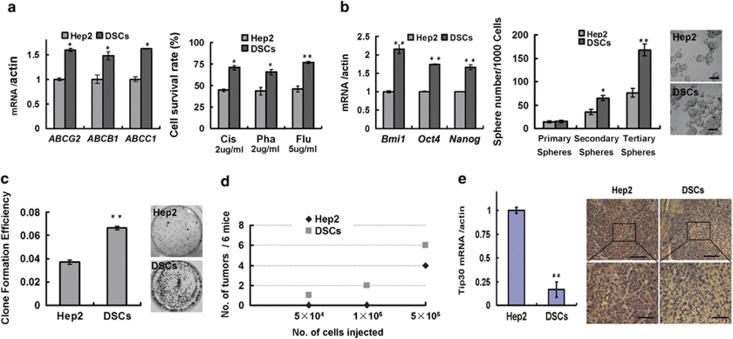
DSCs exhibit enhanced self-renewal, chemoresistance, tumourigenic ability and decreased TIP30 expression. (**a**) Comparison of ABC transporter mRNA levels (ABCG2, ABCB1 and ABCC1) and cell survival rates between DSCs and Hep2 cells following exposure to chemotherapeutic agents (cisplatin, pharmorubicin or 5-fluorouracil) for 72 h (*n*=3). (**b**) mRNA levels of the self-renewal markers (Bmi1, Oct4 and Nanog) were detected in the indicated cells (*n*=3). Mammospheres formed during three *in vitro* serial passages were quantified in DSCs and Hep2 cells. Data are reported as the number of mammospheres formed/1000 seeded cells±S.E. Bars denote the S.E. (*n*=5). Scale bar, 100 *μ*m. (**c**) An aliquot of 1 × 10^4^ DSCs or Hep2 cells in single-cell suspensions were plated in 10-cm-diameter dishes. Clones containing >50 cells were counted, and the clone formation efficiency (CFE) is represented as the ratio of the clone number to the plated cell number. Bars denote the S.E. (*n*=3). (**d**) Aliquots of 5 × 10^4^, 1 × 10^5^ or 5 × 10^5^ DSCs or Hep2 cells were injected subcutaneously into nude mice (*n*=6 mice/group) and tumour incidence was monitored in each group. (**e**) Quantitative reverse transcription–polymerase chain reaction analysis was performed to assess TIP30 mRNA levels in DSCs and Hep2 cells (*n*=3). Representative photographs of immunohistochemical staining for TIP30 expression were taken. (Upper: scale bar, 100 *μ*m; lower: scale bar, 50 *μ*m). *P<0.05 and **P<0.01

To evaluate the drug-resistant abilities of DSCs, we first measured mRNA levels of three ATP-binding cassette transporters (ABCG2, ABCB1 and ABCC1), which have a crucial role in the development of multidrug resistance.^24,25^ Significantly higher mRNA levels of these transporters were found in DSCs than that in Hep2 cells (Figure 1a, left). We then tested their resistance to three common chemotherapeutic agents, that is, cisplatin, pharmorubicin and 5-FU. DSCs were more resistant to the three tested chemotherapeutic agents compared with Hep2 cells (Figure 1a, right).

As DSCs may have stem-like properties,^4^ we examined DSC's self-renewal ability. The expression of self-renewal markers such as Bmi1, Oct4 and Nanog was significantly higher in DSCs than in Hep2 cells (Figure 1b, left). As CD133 is a marker present in a subset of cancer stem-like cells in patients with laryngeal carcinoma,^26^ we measured CD133 expression in DSCs and Hep2 cells by flow-assisted cell sorting analysis. The percentage of CD133-positive cells was much higher in freshly isolated DSCs than in Hep2 cells (Supplementary Figure S2). Sphere generation is an *in vitro* assay of self-renewal potential^9^. Although no obvious difference in primary sphere formation was detected between DSCs and Hep2 cells, the number of spheres formed by DSCs was about two times more than that of Hep2 cells during secondary and tertiary sphere formation (Figure 1b, right). Clone formation assays revealed that DSCs proliferated much faster than that of Hep2 cells (Figure 1c). We further measured the efficacy of xenograft formation by DSCs and Hep2 cells. When 5 × 10^4^ or 1 × 10^5^ cells were injected into BALB/c mice, tumour nodes were found in one and two DSC-xenografted mice, respectively, but no tumour nodes were found in Hep2-xenografted mice (Figure 1d).

TIP30 is downregulated in several human tumours,^15,17,18,22^ and TIP30 overexpression may increase the sensitivity of HCC cells to chemotherapeutic drugs such as 5-FU.^18^ Sorafenib may regulate TIP30 expression to inhibit tumour metastasis through the Jun-activated kinase and signal transducer and activator of transcription 3 signalling pathways.^23^ We measured TIP30 expression in DSCs. We isolated total RNA in fresh tumour tissues from cisplatin- or PBS-treated Hep2-xenografted mice, as shown in Supplementary Figure S1. TIP30 expression was significantly lower in DSCs than in Hep2 cells (Figure 1e, left). The decreased expression of TIP30 in DSCs was further confirmed by immunohistochemical staining (Figure 1e, right).

Collectively, these data show that TIP30 expression decreased in DSCs and that the reduced TIP30 expression may be involved in chemoresistance of LSCC cells.

### TIP30 negatively regulates stem-like properties and chemoresistance of DSCs *in vitro*

**Figure 2:**
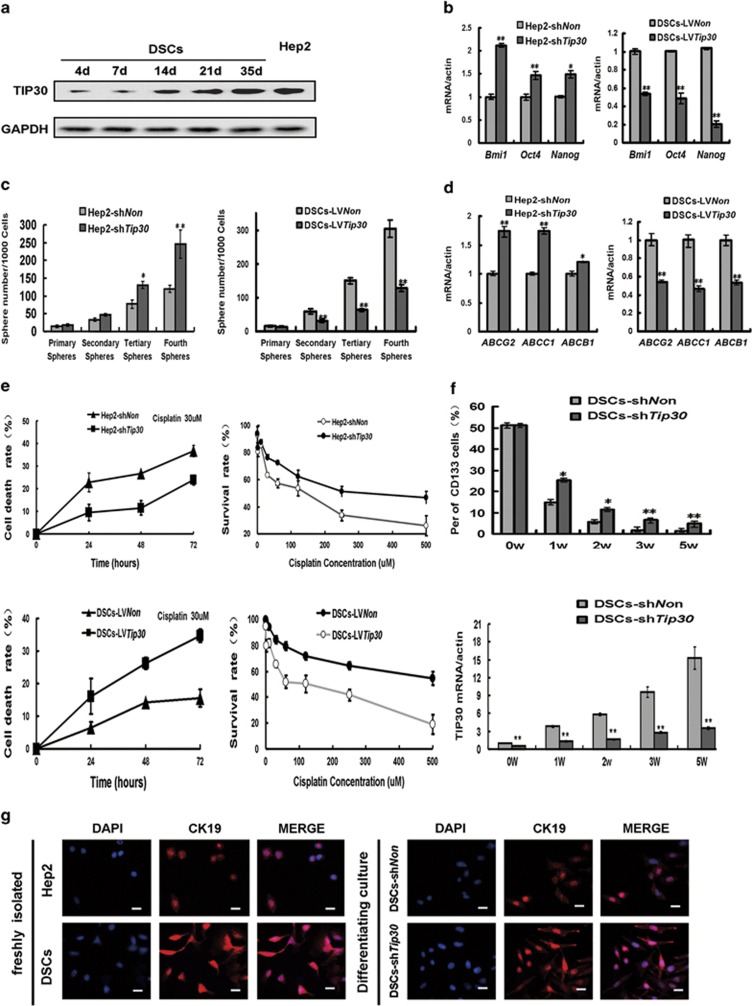
TIP30 increases gradually in DSCs by adherent growth and is negatively involved in the regulation of self-renewal and chemotherapeutic resistance in laryngeal carcinoma cells. (**a**) Western blot was performed to assess TIP30 protein levels in Hep2 cells and DSCs at different time points under adherent conditions. (**b**) Hep2 cells were infected with short sh*Tip30*, and DSCs were infected with LV*Tip30* for 5 days. Comparison of self-renewal marker (Bmi1, Oct4 and Nanog) mRNA levels among Hep2-sh*Tip30*, DSCs-LV*Tip30* and control cells (*n*=3). (**c**) Mammospheres that formed during four *in vitro* serial passages were quantified in the indicated cells. Data are reported as the number of mammospheres formed/1000 seeded cells±S.E. Bars denote the S.E. (*n*=5). (**d**) ABC transporter mRNA levels (ABCG2, ABCB1 and ABCC1) were detected in the indicated cells (*n*=3). (**e**) Left: time–response survival curves of described cells treated with 30 *μ*M cisplatin; right: dose–response survival rate curves in the cells described in (**b**) were generated after exposure to various cisplatin concentrations for 72 h. Data are presented as means±S.D. from three independent experiments. (**f**) Flow-assisted cell sorting analysis was performed to determine CD133 expression at different time points in DSCs-sh*Tip30* and control cells in adherent culture. Corresponding TIP30 mRNA levels were also detected. Data are presented as means±S.D. from three independent experiments. (**g**) Cytokeratin 19 immunofluorescence analysis in freshly isolated DSCs *versus* Hep2 cells (left) and in DSC-sh*Tip30 versus* DSC-sh*Non* under differentiating conditions (right). DAPI was used to stain nuclei. Scale bar, 50 *μ*m. *P<0.05 and **P<0.01

TIP30 expression increased gradually after DSCs were cultured under differentiating conditions, indicating that reduced TIP30 expression may be important for DSCs to maintain an undifferentiated status (Figure 2a). To test whether TIP30 expression is important for self-renewal potential in DSCs, the *Tip30* gene was either introduced into DSCs or silenced by RNA interference in Hep2 cells. Self-renewal-associated transcription factors such as Bim1, Oct4 and Nanog increased significantly when TIP30 was silenced in Hep2 cells and decreased when TIP30 was introduced into DSCs (Figure 2b and Supplementary Figure S3A). The sphere formation assay revealed that silencing TIP30 led to enhanced sphere formation in Hep2 cells, whereas introducing TIP30 inhibited sphere formation in DSCs (Figure 2c and Supplementary Figure S3B). Cell proliferation was significantly enhanced in TIP30-silenced Hep2 cells and diminished in TIP30-overexpressed DSCs (Supplementary Figure S4). Furthermore, G0/G1-phase cell cycle arrest was observed in DSC lentivirus (LV) *Tip30*. Conversely, the proliferation index (percentage of cells in the S and G2 phases) was confirmed to increase significantly after TIP30 silencing in Hep2 cells (Supplementary Figure S5).

To further explore the effect of TIP30 on chemoresistance in LSCC cells, we measured the ABCG2, ABCB1 and ABCC1 mRNA levels. Silencing TIP30 significantly enhanced the expression of ABCG2, ABCB1 and ABCC1 in Hep2 cells, whereas introducing TIP30 into DSCs decreased the expression of these genes (Figure 2d and Supplementary Figure S3A). Moreover, silencing TIP30 in Hep2 cells increased resistance to chemotherapeutic drugs such as cisplatin. When DSCs were infected with LV*Tip30*, their chemotherapeutic sensitivity increased significantly (Figure 2e).

CD133 is an LSCC cell-surface marker, and CD133^+^ cells have enhanced the potential for self-renewal and tumourigenesis.^26^ Freshly isolated DSCs contained about 51.23% of CD133^+^ cells (Supplementary Figure S2). However, after 3 weeks of culture in differentiation medium, accompanied by increased TIP30 expression, most of the CD133^+^ DSCs differentiated into CD133^−^ (Figure 2f). Downregulation of TIP30 in DSCs enhanced the percentage of residual CD133^+^ cells under differentiating conditions (Figure 2f). Cytokine 19 (CK19) is a marker of cancer stem-like cells in head and neck carcinoma and increases significantly in side-population cells compared with that in non-side-population cells.^27^ Therefore, we measured CK19 expression in freshly isolated DSCs and Hep2 cells by immunofluorescence staining. The results showed that CK19 expression was significantly higher in DSCs than in Hep2 cells. Downregulation of TIP30 in DSCs attenuated the elimination of CK19 under differentiating conditions (Figure 2g). Therefore, low TIP30 expression may be required to maintain stem-like properties in DSCs.

Taken together, our results indicate that decreased TIP30 expression is involved in regulating stem-like properties and chemoresistance in LSCC.

### TIP30 negatively regulates tumourigenesis and chemoresistance of LSCC cells *in vivo*

**Figure 3:**
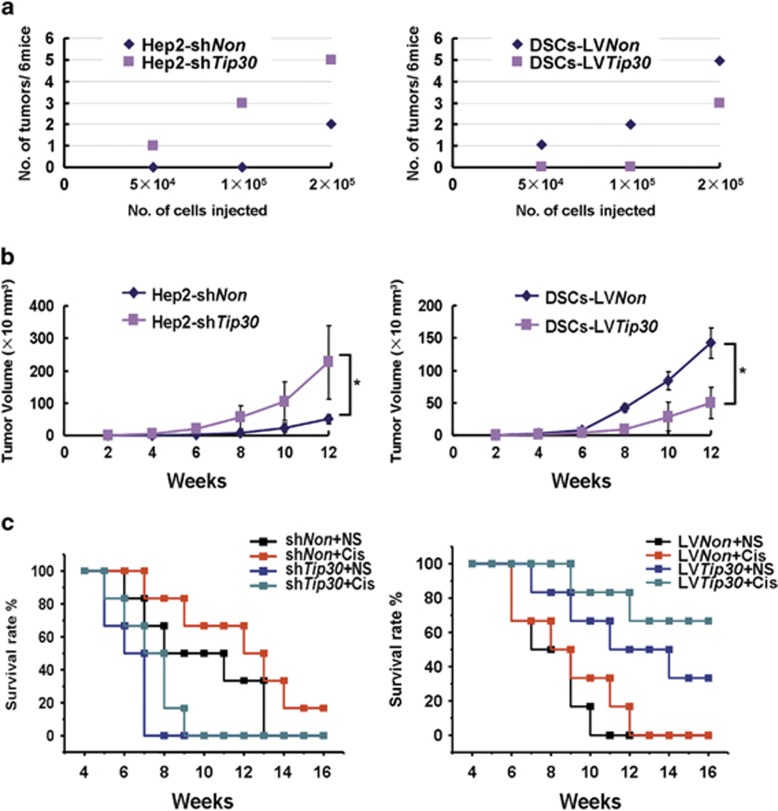
TIP30 exerts an inhibitory effect on tumourigenesis and chemoresistance *in vivo*. (**a**) Aliquots of 5 × 10^4^, 1 × 10^5^ or 2 × 10^5^ Hep2-sh*Tip30*, DSC-LV*Tip30* or control cells were injected subcutaneously into nude mice (*n*=6 mice/group) and tumour incidence was monitored in each group. (**b**) Tumour size was monitored once every 2 weeks with callipers 2 weeks after orthotopic xenograft transplantation of 1 × 10^6^ of the indicated cells in nude mice (*n*=6 mice/group), and tumour volume curves were prepared (*P<0.05). (**c**) Survival rates of nude mice (*n*=6 mice/group) with 5 × 10^6^ Hep2-sh*Non*, Hep2-sh*Tip30*, DSCs-LV*Non* or DSCs-LV*Tip30* subcutaneously implanted, combined with an intraperitoneal injection of cisplatin (2 *μ*g/g) or sodium chloride two times per week

We examined the tumourigenicity of LSCC cells *in vivo* to further determine the effect of TIP30 on stem-like cell properties. Hep2 cells depleted of TIP30 showed enhanced *in vivo* tumour-forming abilities, whereas introducing TIP30 into DSCs significantly attenuated their tumourigenicity according to a serial dilution assay in nude mice (Figure 3a). Moreover, silencing TIP30 significantly enhanced the growth of Hep2 cells *in vivo*, whereas forced TIP30 expression significantly decreased the growth of DSCs (Figure 3b). These data suggest that TIP30 exerts a negative effect on tumourigenesis in LSCC. The same results were obtained in Hep2 cells infected with a second set of *Tip30*-short hairpin RNA (shRNA) LV (Supplementary Figures S3C and D).

LSCC cells were implanted subcutaneously in nude mice combined with cisplatin or sodium chloride treatment as a control to investigate the effect of TIP30 on chemotherapy *in vivo*. Silencing TIP30 significantly decreased the survival of Hep2-xenografted mice (*P=*0.023) and the chemotherapeutic effect of cisplatin (*P=*0.006) (Figure 3c, left). In contrast, introducing TIP30 into DSCs cells significantly enhanced the survival of DSC-xenografted mice (*P=*0.021) and the chemotherapeutic effect of cisplatin (*P=*0.007) (Figure 3c, right).

These results clearly suggest that TIP30 expression is important for treating LSSC with chemotherapeutic drugs.

### AKT/GSK-3 ***β** / **β***-catenin signalling is required for TIP30 to regulate self-renewal, chemoresistance and proliferation of LSCC cells

**Figure 4:**
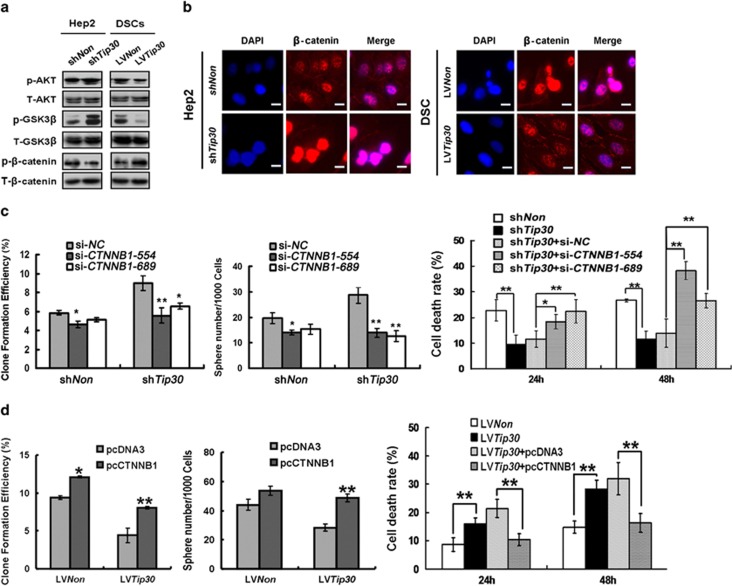
The AKT/*GSK-3β/β*-catenin pathway is required for TIP30-induced inhibition of self-renewal, chemoresistance and proliferation. (**a**) Western blot was performed to assess phosphorylated and total AKT, GSK-3*β* and *β*-catenin expression in Hep2-sh*Tip30*, DSC-LV*Tip30* and control cells. (**b**) *β*-Catenin immunofluorescence analysis for the cells described in (**a**). DAPI was used to stain the nuclei. Scale bar, 25 *μ*m. (**c**) Clones that formed and the spheres that were generated in Hep2-sh*Non* or Hep2-sh*Tip30* cells transfected with siRNA targeting *β*-catenin (si-*CTNNB1-554* or si-*CTNNB1-689*) or scrambled siRNA (si-*NC*). Cell death rates were tested in Hep2-sh*Non*, Hep2-sh*Tip30* and Hep2-sh*Tip30* transfected with si-*CTNNB1-554*, si-*CTNNB1-689* or si-*NC*, combined with cisplatin treatment (30 *μ*M) for 24 or 48 h, respectively. Bars denote the S.E. (*n*=5). (**d**) Clones that formed and spheres that were generated in DSCs-LV*Non* or DSCs-LV*Tip30* transfected with pcDNA3 or pc*CTNNB1*. Cell death rates were tested in DSCs-LV*Non*, DSCs-LV*Tip30* and DSCs-LV*Tip30* transfected with pcDNA3 or pc*CTNNB1*, combined with cisplatin treatment (30 *μ*M) for 24 or 48 h, respectively. Bars denote the S.E. (*n*=5). *P<0.05 and **P<0.01

Loss of TIP30 improves epidermal growth factor receptor (EGFR) activity, which leads to the activation of AKT and extracellular-regulated kinase (ERK)1/2 in human lung adenocarcinoma and mammary tumours.^28,29^ AKT/glycogen synthase kinase-3*β* (GSK-3*β*)/*β*-catenin signalling has important roles in the development of multiple tissues by regulating cell self-renewal, proliferation, differentiation and movement.^30,31^ Here, we found that downregulating TIP30-activated AKT caused subsequent GSK-3*β* Ser9 phosphorylation (an inactive form of GSK-3*β*) and less *β*-catenin Thr41/Ser45 phosphorylation (recognized by proteasome for degradation) in Hep2 cells. In contrast, overexpressing TIP30 greatly attenuated AKT activation, reduced GSK-3*β* Ser9 phosphorylation and enhanced *β*-catenin Thr41/Ser45 phosphorylation in DSCs (Figure 4a). Thus, downregulation of TIP30 could enhance *β*-catenin stability and overexpression of TIP30 could promote *β*-catenin degradation. Indeed, TIP30 knockdown increased total *β*-catenin expression in Hep2 cells, whereas enforced expression of TIP30 in DSCs resulted in decreased total *β*-catenin expression (Supplementary Figure S6). Consistently, nuclear accumulation of *β*-catenin was observed in TIP30-silenced Hep2 cells, whereas forced expression of TIP30 resulted in cytoplasmic distribution of *β*-catenin in DSCs (Figure 4b). Moreover, a significant negative correlation was observed between TIP30 expression and p-AKT levels (*r*=0.452, *P*<0.001) or *β*-catenin distribution (*r*=0.454, *P*<0.001) in paraffin-embedded LSCC tissues by immunohistochemistry analysis (Supplementary Figure S7 and Supplementary Table S1).

Inhibiting *β*-catenin signalling with small interfering RNA (siRNA) significantly inhibited the TIP30 deficiency-induced enhanced ability for sphere formation, proliferation and resistance to cisplatin (Figure 4c). In contrast, overexpressing *β*-catenin attenuated the TIP30-induced inhibitory effect on sphere formation, proliferation and resistance to cisplatin in DSCs (Figure 4d). Therefore, AKT/GSK-3*β/β*-catenin signalling is required for TIP30 to regulate self-renewal, chemoresistance and proliferation of LSCC cells.

### Decreased TIP30 expression is related to poor prognosis in patients with laryngeal carcinoma

**Figure 5:**
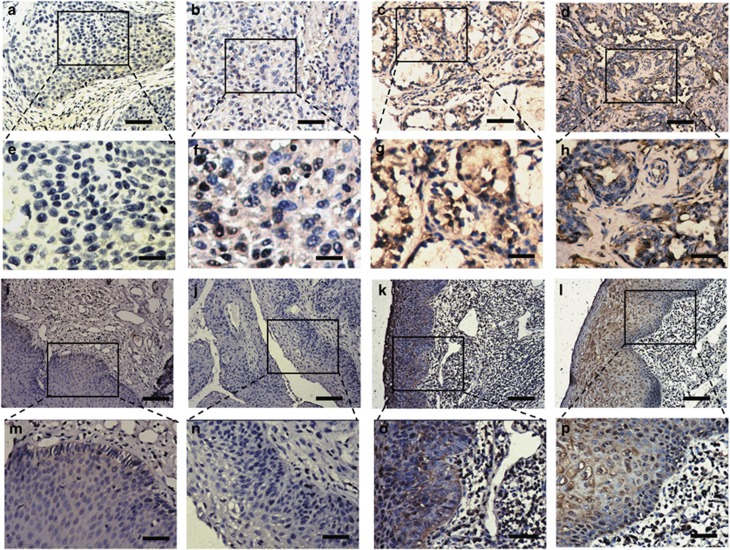
Representative TIP30 immunohistochemical staining in human laryngeal carcinoma tissues and adjacent non-carcinoma tissues. Negative TIP30 expression in tumour tissues (**a**, **b**, **e** and **f**). Positive TIP30 expression in tumour tissues (**c**, **d**, **g** and **h**). Negative TIP30 expression in adjacent non-carcinoma tissues (**i**, **j**, **m** and **n**). Positive TIP30 expression in adjacent non-carcinoma tissues (**k**, **l**, **o** and **p**). (**a**–**d** and **i**–**l**) Scale bar, 100 *μ*m; (**e**–**h** and **m**–**p**) scale bar, 50 *μ*m

TIP30 expression was examined in 105 laryngeal carcinomas and adjacent non-tumour tissues using anti-human TIP30 antibody. TIP30 immunostaining was detected in the cell cytoplasm. The proportion of negative TIP30 expression was higher in tumour tissues than in non-tumour tissues (Figure 5). Minimal TIP30 staining of tumour cells was found in 46 cases (43.8%), whereas minimal TIP30 staining in non-tumour cells was detected in 27 cases (25.7% *P*=0.006). To further validate the immunostaining results, we examined TIP30 expression in eight freshly isolated laryngeal carcinomas and non-tumour tissues by western blot analysis. As shown in Supplementary Figure S8, TIP30 expression in the adjacent non-tumour tissues was significantly higher than that in tumour tissues.

Based on the TIP30 expression results in tumour cells, 105 patients were divided into a high-expressing (*n*=59) and low-expressing group (*n*=46), and the correlations between TIP30 expression and clinicopathologic features were analysed. TIP30 was inversely correlated with T stage (*P<*0.001). However, other clinical characteristics, including age, sex, lymph node metastasis, clinical stage, tumour site and histological differentiation, were not significantly related to TIP30 expression (Table 1).

**Figure 6:**
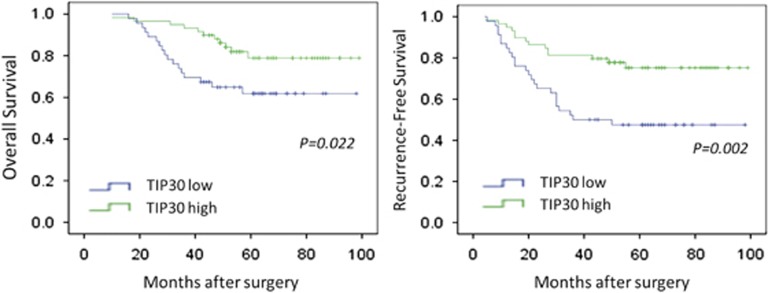
Low TIP30 expression predicts a poor prognosis in patients with laryngeal carcinoma. Kaplan–Meier survival curves showing overall (left) and recurrence-free survival rates (right) of 105 patients with laryngeal carcinoma between the TIP30-high *versus* TIP30-low-expressing groups (log-rank test)

The potential associations between TIP30 immunostaining and overall survival (OS) and recurrence-free survival (RFS) were retrospectively evaluated in these patients. A Kaplan–Meier analysis showed that OS (*P*=0.022) and RFS (*P*=0.002) were significantly better among patients with high TIP30 staining compared with those with low TIP30 staining (Figure 6). A univariate analysis showed that sex, age, T stage and clinical stage had no prognostic significance for RFS and OS. However, tumour site, histologic differentiation and TIP30 expression were predictors of RFS and OS, whereas lymph node metastasis only had prognostic significance for RFS (Supplementary Table S2). In a multivariate analysis, we found that lymph node metastasis, histologic differentiation and TIP30 expression were independent prognostic factors for RFS. Histologic differentiation and TIP30 expression also had independent prognostic value for OS (Supplementary Table S3).

We conclude that decreased expression of TIP30 may be a prognostic indicator for poor survival in patients with laryngeal carcinoma. These results confirmed that TIP30 was an independent variable, and, importantly, suggest that TIP30 negatively represents a significant risk factor for patient survival.

## Discussion

Despite the LSCC treatment strategies that have been developed in the past two decades,^32, 33, 34^ satisfactory therapeutic outcomes and survival rates of patients with LSCC have not improved significantly.^35^ Drug resistance and recurrence of malignancy are responsible for the uncertain therapeutic effects.

Drug-resistant tumour cells can be obtained by *in vivo* or *in vitro* administration of chemotherapeutic drugs to kill proliferating cells.^36, 37, 38^ These DSCs have the properties of stem-like cells, with self-renewal ability, and can be used for further research on drug resistance.^36, 37, 38^ To better understand the mechanisms of chemoresistance in LSCC cells, we generated DSCs, a highly malignant laryngeal cancer cell line, by treating Hep2 cells with cisplatin *in vivo.*

Our results showed that DSCs displayed cancer stem-like properties, including enhanced sphere formation, chemoresistance, tumourigenesis and a CD133^+^ population. DSCs had about 20-fold more tumourigenic ability than the parent cells and much higher expression of self-renewal markers (Bmi1, Oct4 and Nanog). Moreover, we found that *Tip30*, a novel tumour suppressor gene, was hardly expressed at the mRNA and protein levels in freshly isolated DSCs, indicating that TIP30 may have an important role in drug resistance and self-renewal in patients with LSCC.

Previous reports have suggested that TIP30 has an important role in many malignancies, including liver cancer, lung cancer, pancreatic cancer and breast cancers.^22,39, 40, 41, 42^ Loss of TIP30 causes spontaneous development of liver, lung and mammary cancers.^15,28,43^ We reported previously that TIP30 may execute its antitumour effect by stabilising p53 mRNA and promoting apoptosis.^41^ Ectopic expression of TIP30 in HCC cells enhances p53 expression and decreases Bcl-2/Bcl-xL expression by which TIP30 increased sensitivity of the cytotoxic drug 5-FU and prolonged survival of nude mice bearing subcutaneously established HCC tumours.^18^ In the present study, we found that depleting TIP30 negatively regulated the resistance of LSCC to chemotherapeutic drugs. The mRNA levels of ABC half-transporters, which are believed to be important regulators of drug resistance, decreased significantly in TIP30-overexpressed cells and increased in TIP30-silenced cells. Knocking down TIP30 made Hep2 cells more resistant to cisplatin, whereas forced expression of TIP30 made DSC cells sensitive to cisplatin. Most importantly, overexpressing TIP30 in combination with cisplatin treatment significantly increased survival of LSCC-xenografted mice, whereas TIP30 knockdown significantly attenuated the therapeutic effects of cisplatin in LSCC-xenografted mice.

Previous studies have shown that TIP30 may be involved in regulating properties of stem cells or precursor cells. Deleting *Tip30* leads to expansion of bronchoalveolar stem/progenitor cells in *Tip30*-knockout BALB/c mice,^40^ whereas overexpressing TIP30 in a rat oligodendrocyte precursor cell line inhibits its differentiation.^44^ Here, we found that forced expression of TIP30 attenuated the stem-like properties of DSCs by inhibiting AKT/GSK-3*β/β*-catenin signalling. A previous report suggested that loss of TIP30 improves EGFR activity, which leads to upregulated p-AKT and pERK1/2 in human lung adenocarcinoma and mammary tumours.^28,29^ p-AKT phosphorylates GSK3*β* to regulate activation of *β*-catenin.^45,46^ *β*-Catenin is a multifunctional protein with a central role in various diseases including cancer, and abnormal activation of *β*-catenin signalling is related to drug resistance. Additionally, nuclear localisation of *β*-catenin is a characteristic of stem-like cell populations in cancers that are capable of initiating tumour formation.^47^ Our data revealed that silenced TIP30 expression accompanied by AKT and GSK-3*β* phosphorylation attenuated the phosphorylated *β*-catenin level and led to nuclear accumulation of *β*-catenin. Inhibiting *β*-catenin significantly inhibited TIP30 deficiency-induced enhanced abilities for sphere formation, proliferation and chemoresistance. In contrast, the adverse effects of upregulated *β*-catenin were also confirmed. Thus, TIP30 deficiency-induced activation of the AKT/GSK-3*β/β*-catenin pathway is critical for chemoresistance, self-renewal and proliferation of LSCC cells.

In addition, we assessed TIP30 expression on a larger scale in clinical samples from patients with laryngeal carcinoma and adjacent tissues for the first time. Low TIP30 expression was found in laryngeal carcinoma and was significantly associated with T stage. The importance of the TIP30 protein in the development of laryngeal carcinoma was underscored by its low-level expression in laryngeal carcinoma. We suggest that decreased TIP30 expression may be a prognostic indicator of poor survival for patients with laryngeal carcinoma because of the higher recurrence and death rates.

In conclusion, we found that TIP30 regulates the potential for chemoresistance and self-renewal in LSCC cells. These effects of TIP30 were related to the regulation of the AKT/GSK-3*β/β*-catenin pathway. Moreover, our data reveal that TIP30 decreased in LSCC tumour tissues and was associated with progression and prognosis. Taken together, these results suggest that TIP30 may be a useful candidate to improve chemotherapeutic results and serve as a marker for prognosis in patients with LSCC.

## Materials and Methods

### Cell culture and LV

Hep2 cells were obtained from the Institutes for Biological Sciences (Shanghai, People's Republic of China). The cells were grown in 1640-DMEM (Dulbecco's modified Eagle's medium) with 10% foetal bovine serum at 37 °C in a humidified atmosphere of 5% CO_2_ in air. An LV encoding shRNA targeting *Tip30* was infected as reported previously.^48^ The double-stranded oligo DNAs for sh*Tip30*-2 were: top strand, 5′-CCGGCCTCTAAAGGAGCTGATAAATCTCGAGATTTATCAGCTCCTTTAGAGGTTTTTg-3′ and bottom strand, 5′-CCGGGCAGAATAAATCCGTCTTTATCTCGAGATAAAGACGGATTTATTCTGCTTTTTg-3′.

### Plasmids, siRNA and transfection

pcCTNNB1 was purchased from Addgene (Cambridge, MA, USA). An siRNA specific to *CTNNB1* and control siRNA were purchased from Shanghai GenePharma (Shanghai, People's Republic of China). The cells were transfected using DharmaFect (Thermo Fisher Scientific, Rockford, IL, USA) according to the manufacturer's instructions. A total of 2 M of the siRNA duplex was used for each transfection. The sequences were as follows: for *CTNNB1*-554, sense –5′-GCUGCUAUGUUCCCUGAGATT-3′ and antisense – 5′-UCUCAGGGAACAUAGCAGCTT-3' for *CTNNB1*-689, sense – 5′-GCAGUUGUAAACUUGAUUATT-3' and antisense – 5′-UAAUCAAGUUUACAACUGCTT-3'.

The efficiency of LV infection and small interfering and plasmid transfection is shown in Supplementary Figure S9.

### Cell sphere culture

Cells were resuspended in serum-free DMEM-F12 medium (Invitrogen, Carlsbad, CA, USA) supplemented with 4 *μ*g/ml insulin (Sigma-Aldrich, St. Louis, MO, USA), 20 ng/ml EGF (Peprotech, Rocky Hill, NJ, USA), 20 ng/ml basic fibroblast growth factor (Peprotech), B27 (1 : 50 Invitrogen) and 0.4% bovine serum albumin (BSA). The cells were plated onto ultralow attachment plates (Corning, New York, NY, USA). A 200-*μ*l aliquot of 0.8% BSA-F12 medium containing EGF and insulin was added every 3 days. The number of spheroids was measured under a microscope 14 days after seeding. The spheres were collected by centrifugation for serial passage, dissociated into single cells with trypsin and cultured under the conditions described above.

### Cell viability and chemoresistance assay

Cells were detached, counted and seeded on 96-well plates at 5000 cells per well. The chemotherapeutic agent obtained from Changhai Hospital was added to the medium at a particular concentration 24 h later. The medium was removed, and the plates were washed with PBS two times after the designated time point. Cell viability was determined with an MTS assay reagent (CellTiter 96 AQueous one Solution Cell Proliferation Assay; Promega, Madison, WI, USA) according to the manufacturer's protocol.

### Clone formation

A single-cell suspension of 10 000 cells was plated in a 10-cm-diameter dish and cultured in 1640-DMEM for 10 days. After most of the cell clones had expanded to >50 cells, they were washed two times with PBS and dyed with crystal violet for 15 min at room temperature. After washing out the dye, clones containing >50 cells were counted. Clone formation efficiency (CFE) was the ratio of the clone number to the plated cell number.

### Tumour xenograft mouse model

Six-week-old male BALB/c nude mice were purchased from the Shanghai Experimental Animal Centre of the Chinese Academy of Sciences (Shanghai, People's Republic of China) and were maintained under specific pathogen-free conditions. The animal care and experimental protocols were conducted in accordance with the guidelines of the Shanghai Medical Experimental Animal Care Commission. Aliquots of cells were injected subcutaneously into each mouse for limiting dilution tumour formation. The animals were killed at the indicated time intervals when tumour nodules were identified on the body surface of the mice. If no tumours were found, the mouse was monitored until the second or third time point. An aliquot of 1 × 10^6^ cells was injected subcutaneously into the mice to evaluate tumour-forming ability, and the mice were monitored once every 2 weeks for palpable tumours. Tumour size was measured with callipers, and tumour volume was calculated according to the following formula: larger diameter × (smaller diameter)^2^/2.

### Patients, immunohistochemistry and scoring

In total, 105 laryngeal carcinoma samples with adjacent non-tumour tissues were obtained from patients who had undergone surgery between 2004 and 2009 at Changhai Hospital (Shanghai, People's Republic of China). Tumour stage was determined according to the NCCN clinical practice guidelines of the oncology – head and neck cancer guidelines. All paraffin-embedded tissue specimens were diagnosed and reconfirmed by two experienced pathologists. Complete follow-up data were available for all patients, and the clinicopathologic characteristics of the patients are summarised in Supplementary Table S4. Patient follow-up was completed in May 2012. Recurrences were confirmed based on typical imaging appearance on computed tomography scans and/or magnetic resonance imaging.

Rabbit anti-human TIP30 (1 : 6000, generated in our lab as reported previously^39^), rabbit anti-human p-AKT (1 : 50; Cell Signaling Technology, Danvers, MA, USA) and rabbit anti-human *β*-catenin (1 : 300; Cell Signaling Technology) were used to detect TIP30, p-AKT and *β*-catenin expression. The immunohistochemistry protocols have been described elsewhere.^39^ One set of samples was incubated with non-immune rabbit IgG (1 : 150; Santa Cruz Biotechnology, Santa Cruz, CA, USA) instead of primary antibody as an antibody control. The staining evaluation was performed independently by two experienced pathologists. Immunostaining intensity was semiquantitatively estimated according to signal intensity and distribution. Briefly, the mean percentage of positive tumour cells was determined in at least five areas at × 400 magnification and assigned to one of the following five categories: 0, <5% 1, 5–25% 2, 25–50% 3, 50–75% and 4, >75%. Immunostaining intensity was scored as follows: 1, weak; 2, moderate and 3, intense. The predominant pattern of the tumours that showed heterogeneous staining was considered for scoring. The percentage of positive tumour cells and staining intensity were multiplied to produce a weighted score for each case. Tissues with an immunohistochemical score ≤4 were considered low expressing, and those >4 were considered high expressing. Carcinoma cells staining positively for membranous *β*-catenin were classified as normally expressing, whereas positive staining of the cytoplasm or the nuclei was considered abnormal *β*-catenin expression. The use of all human samples and the experimental procedures for this study were reviewed and approved by the university and hospital ethics committees.

### Statistical analysis

Statistical analyses were carried out using SPSS 16.0 for Windows software (SPSS Inc., Chicago, IL, USA). *P*-values for dichotomous variables were based on Pearson's *χ*^2^-test. Continuous variables were analysed with Student's *t*-test. Spearman's rank correlation test was used to analyse bivariate correlations. The Kaplan–Meier method was used to determine survival probability, and differences were assessed by the log-rank test. OS was defined as the interval between surgery and death or between surgery and the last observation point. Data of surviving patients were censored at the last follow-up. RFS was defined as the interval between the date of surgery and the date of diagnosis of any type of relapse. Univariate and multivariate analyses were based on the Cox proportional hazards regression model. All statistical tests were two-sided, and *P*<0.05 was considered significant.

## Figures and Tables

**Table 1:** The associations of TIP30 expression with clinicopathologic characteristics in laryngeal carcinoma patients

**Feature**	**Low expression of TIP30 (n=46)**	**High expression of TIP30 (n=59)**	***P*-value**
*Gender*			
Male	41	51	0.678
Female	5	8	
			
Age (*years)*			
≤60	24	22	0.127
≤60	22	37	
			
*T stage*			
T1–T2	22	51	0.000
T3–T4	24	8	
			
*Lymph node metastasis*			
N−	25	37	0.387
N+	21	22	
			
*Clinical stage*			
I–II	22	32	0.514
III–IV	24	27	
			
*Tumour site*			
Subglottic	21	20	0.142
Glottic	17	33	
Supraglottic	8	6	
Histologic differentiation			
G1–G2	24	28	0.632
G3–G4	22	31	

## Abbreviations

ABC: ATP-binding cassette half-transporter
CFE: clone formation efficiency
CK19: cytokine 19
DSC: drug-selected cell
GSK-3*β*: glycogen synthase kinase-3*β*
LSCC: laryngeal squamous cell carcinoma
OS: overall survival
RFS: recurrence-free survival
