# Supplementary figures and images for: Contribution of TIP30 to chemoresistance in laryngeal carcinoma
Source: Cell Death Dis. 2014 Oct 16;5(10):e1468–. doi: 10.1038/cddis.2014.424 (PMC4237250; doi:10.1038/cddis.2014.424)

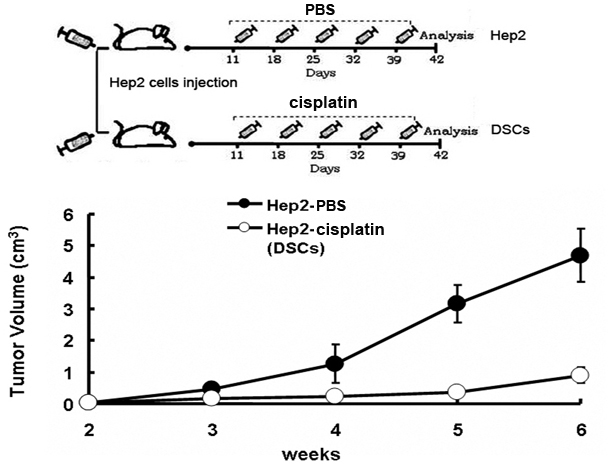

Supplement: Supplementary Figure S1 [file cddis2014424x1.tif]

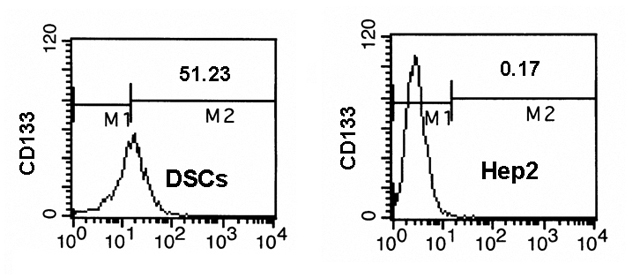

Supplement: Supplementary Figure S2 [file cddis2014424x2.tif]

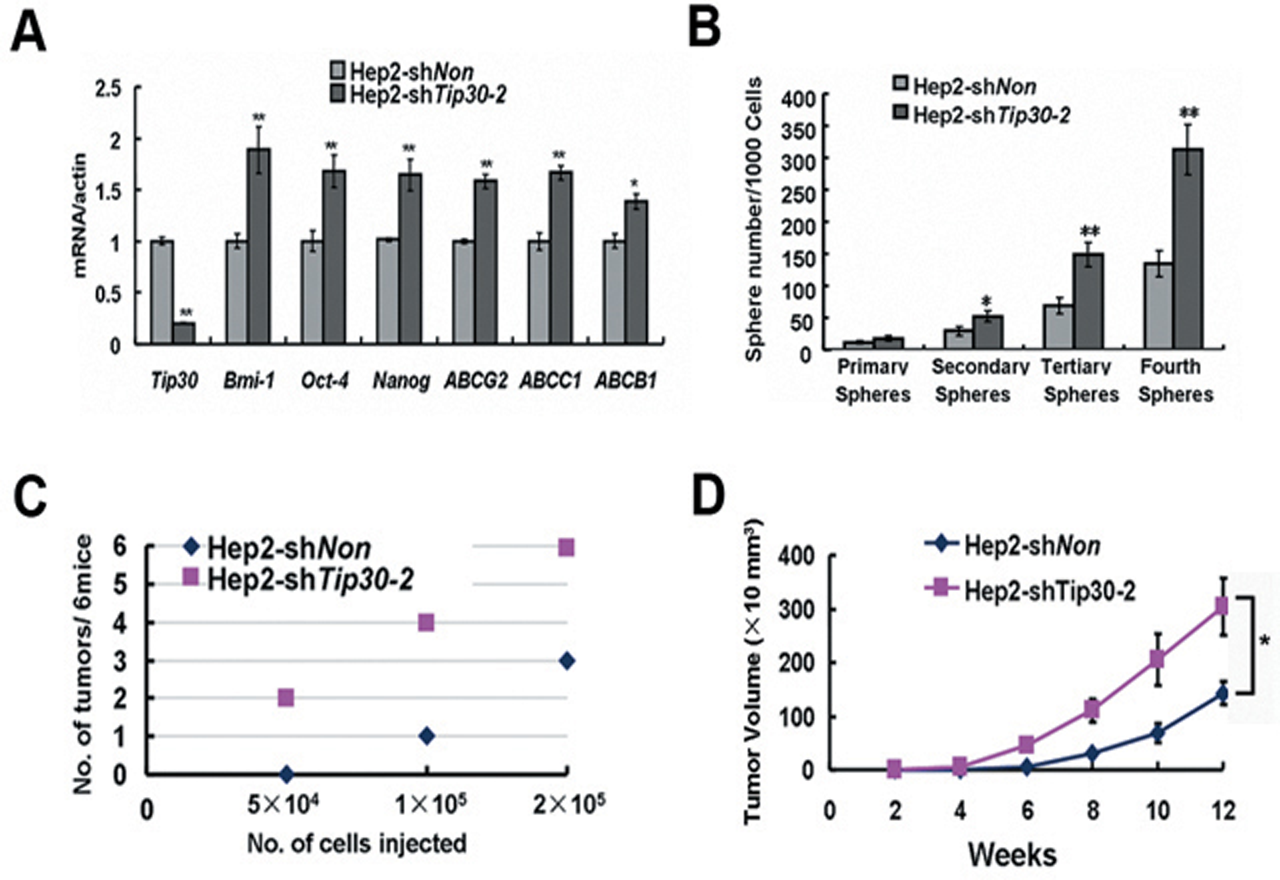

Supplement: Supplementary Figure S3 [file cddis2014424x3.tif]

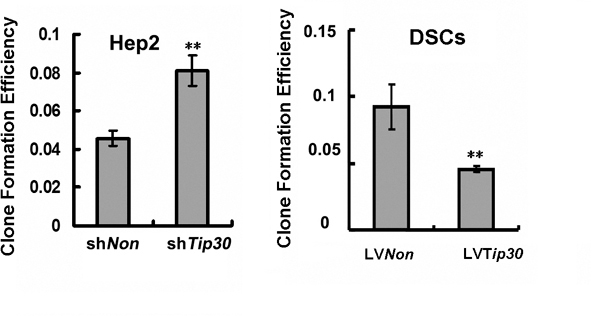

Supplement: Supplementary Figure S4 [file cddis2014424x4.tif]

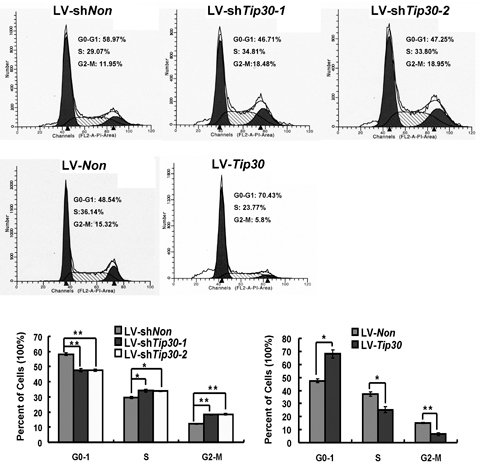

Supplement: Supplementary Figure S5 [file cddis2014424x5.tif]

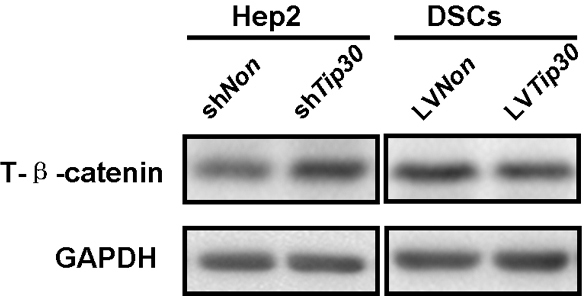

Supplement: Supplementary Figure S6 [file cddis2014424x6.tif]

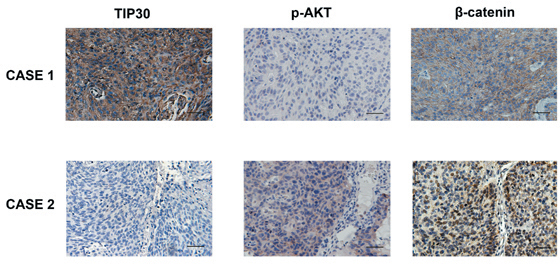

Supplement: Supplementary Figure S7 [file cddis2014424x7.tif]

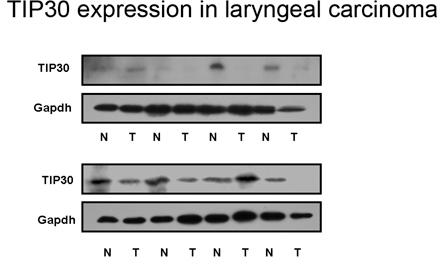

Supplement: Supplementary Figure S8 [file cddis2014424x8.tif]

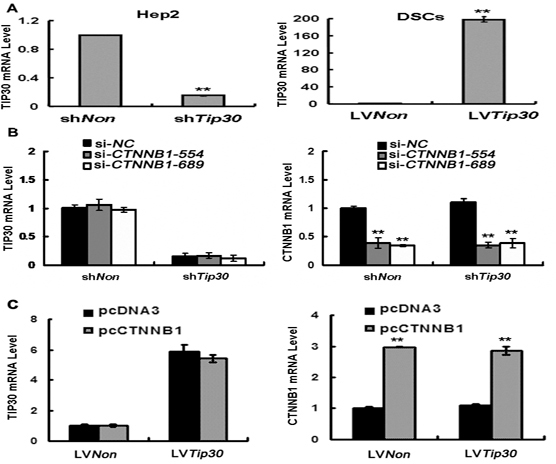

Supplement: Supplementary Figure S9 [file cddis2014424x9.tif]
