# Supplementary material for: Contribution of TIP30 to chemoresistance in laryngeal carcinoma
Source: Cell Death Dis. 2014 Oct 16;5(10):e1468–. doi: 10.1038/cddis.2014.424 (PMC4237250; doi:10.1038/cddis.2014.424)
Supplement: Supplementary Materials and Methods [file cddis2014424x10.doc]

**Supplementary Materials and Methods**

***RNA extraction and reverse transcription PCR. Total RNA was isolated by NucleoSpin RNA II kit (Macherey&Nage, Easton, PA; includes DNase I treatment). First-strand cDNA was generated using the PrimeScript RT reagent Kit (Takara Bio, Tokyo, Japan). Analysis of mRNA levels was performed on a 7500 Fast Real-Time PCR System (Applied Biosystems, Carlsbad, California) with SYBR Green-based real-time PCR. Actin was used as an endogenous control to normalize the amount of total RNA in each sample. The primer sequences are provided as follows (F, forward; R, reverse):***

| **Gene symbol** | **Primer Sequence (5'-3')** |
| --- | --- |
| *TIP30* | F:TCACCTTCGACGAGGAAGCT |
|  | R: GCTCTGCAGACTTCAGACCA |
| *Bmi1* | F: TGGAGAAGGAATGGTCCACTTC |
|  | R: GTGAGGAAACTGTGGATGAGGA |
| *Actin* | F: CGTGGACATCCGTAAAGACC |
|  | R: ACATCTGCTGGAAGGTGGAC |
| *Nanog* | F: GATTTGTGGGCCTGAAGAAA |
|  | R: TTGGGACTGGTGGAAGAATC |
| *Oct4* | F: CTTGCTGCAGAAGTGGGTGGAGGAA |
|  | R: CTGCAGTGTGGGTTTCGGGCA |
| *ABCG2* | F:CAGGTGGAGGCAAATCTTCGT |
|  | R:ACCCTGTTAATCCGTTCGTTTT |
| *ABCC1* | F:CTCTATCTCTCCCGACATGACC |
|  | R:AGCAGACGATCCACAGCAAAA |
| *ABCB1* | F:TTGCTGCTTACATTCAGGTTTCA |
|  | R:AGCCTATCTCCTGTCGCATTA |
| *CTNNB1* | F: ATGTCCAGCGTTTGGCTGAA |
|  | R: TGGTCCTCGTCATTTAGCAGTT |

***Immunofluorescence staining.*** Cells were seeded in 24-well plates fixed with 4% paraformaldehyde for 20-30 minutes at room temperature ,then washed with 1×PBS three times and blocked with blocking solution (0.5% Triton X-100/1% BSA/ 1×PBS ) for 1 hour at room temperature. For immunofluorescence staining, cells were incubated with primary antibodies against β-catenin and CK19 from Cell Signaling Technology (Danvers, MA) in a moist chamber at 4℃ at overnight. They were washed in PBS, blocked again for 30 minutes, and treated with goat anti-rabbit IgG (Invitrogen, Carlsbad, California) at room temperature for 1 hour. After washing in PBS, the cells were then stained with DAPI (Invitrogen, Carlsbad, California). All matched samples were photographed (control and test) using immunofluorescence microscope.

***Western blotting.*** Total cell lysate was prepared in 1×SDS buffer. Protein at the same amount was separated by SDS-PAGE and transferred onto PDVF membranes. After probing with individual antibodies, antigen–antibody complex was visualized by enhanced chemiluminescence’s reagents Supersignal (Pierce Biotechnology, Milwaukee, WI). The following primary antibodies were used: anti-TIP30 (1:2000, generated in our lab as described previously^1^) and anti-GAPDH (Santa Cruz Biotechnology, Santa Cruz, CA) for loading controls ; anti-p-AKT, anti-p-β-catenin (phosphorylation at Thr41/Ser45), anti-p-GSK3β (phosphorylation at Ser-9) (Cell Signaling Technology, Danvers, MA) and anti-T-AKT, anti-T-β–catenin, an ti-T-GSK3β(Cell Signaling Technology, Danvers, MA) for loading controls respectively.

***FACS Analysis.*** For cell surface marker analysis, cells were trypsinized, washed, and re-suspended in PBS supplemented with 1% fetal bovine serum. These cells were incubated at 4℃ for 30 minutes with PE anti-CD133 (MiltenyiBiotec, Auburn, CA) and analyzed with a BD FACS Aria cell sorting system (BD Biosciences, Bedford, MA).

***Cell cycle analysis.*** Cell cycle assays were performed by flow cytometry using propidium iodide (PI) staining. After washing twice with cold PBS, the cells were resuspended in 70% pre-cooled ethanol and fixed overnight at 4℃. Next, the fixed cells were washed with PBS and incubated with RNase A and PI at 37℃ for 30 minutes in the dark. DNA content was measured by fluorescence-activated cell sorting (FACS) using flow cytometry analysis.

**Reference**

1 Tong X, Li K, Luo Z, Lu B, Liu X, Wang T *et al*. Decreased TIP30 expression promotes tumor metastasis in lung cancer. Am J Pathol 2009; 174: 1931-1939.

**Supplementary Figure Legends**

**Figure.S1.Acquisition of drug-selected cells (DSCs).** 2×10^6^ Hep2 cells were subcutaneously injected into the right flank of male BalB/C mice (n=6).These mice were random distributed into 2 groups (n=3 mice/group), which were intraperitoneally injected cisplatin (2ug/g) or PBS respectively every 7 days for 5 cycles. Tumor size was monitored and tumor volume curve was made. 42 days after inoculation, mice were killed by luxation of the neck and cisplatin resistant xenograft was considered to be primary DSCs.

**Figure.S2.**Comparison of CD133 expression between freshly isolated DSCs and Hep2 cells by FACS analysis.

**Figure.S3. Decreased TIP30 prompts in *vitro* stem-like properties and in *vivo* tumorigenesis of Hep2 cells.** (A) Hep2 cells were infected with sh*Tip30-2* for 5 days and lentivirus infection efficiency was assessed. Comparison of mRNA levels of self-renewal markers (Bmi1, Oct4 and Nanog) and ATP-binding cassette transporters (ABCG2, ABCB1, and ABCC1) between Hep2-sh*Tip30-2* and the control cells (n=3). (B) In *vitro* quantification of mammospheres formed during 4 serial passages was conducted in indicated cells. The data was reported as the number of mammospheres formed/1000 seeded cells ± SEM. Bars denote the standard error (n=5) (C) Aliquots of 5×10^4^, 1×10^5^ or 2×10^5^ Hep2-sh*Tip30-2*, or the control cells were injected subcutaneously into nude mice (n=6 mice/group) and tumor incidence was monitored in each group. (D) After two weeks’ orthotopic xenograft transplantation of 1×10^6^ indicated cells in nude mice (n=6 mice/group)**,** tumor size was monitored once two weeks with calipers and tumor volume curve was made. **P* < 0.05, ***P* < 0.01

**Figure.S4.TIP30 is negatively involved in the regulation of proliferation in laryngeal carcinoma cells.** 1×10^4^ Hep2-sh*Tip30*, DSCs-Lv*Tip30* or the control cells in single-cell suspension were plated in 10-cm-diameter dish. Clone that contained >50 cells was counted and CFE was represented as the ratio of the clone number to the planted cell number. (*n* =3). ***P* < 0.01

**Figure.S5. TIP30 knockdown leads to accelerated G1-S transition of cell cycle progression.** Cell cycle analysis by DNA content was performed using flow cytometry in Hep2-sh*Tip30-1*, Hep2-sh*Tip30-2*, DSCs-LV*Tip30* and the control cells. Cell cycle models fit by ModFit LT software and the average percentages of G0/G1, S and G2/M phases were shown. (*n* =3). **P* < 0.05, ***P* < 0.01

**Figure.S6.** Western blot was performed to assess total β-catenin expression in Hep2-sh*Tip30*, DSCs-LV*Tip30* and the control cells.

**Figure.S7. Decreased TIP30 correlates with high p-AKT levels and abnormal β-catenin distribution of LSCC.** 105 LSCC samples were immunostained with anti-TIP30, anti-p-AKT or anti-β-catenin antibody. Representative immunostaining was shown for two patient samples. Scale bar, 50μm.

**Figure.S8.** Western blot was performed to assess TIP30 expression in freshly isolated tumor and corresponding non-tumor tissues of 8 laryngeal carcinomas patients.

**Figure.S9. qRT-PCR was performed to assess lentivirus infection, small RNA interfering and plasmid transfection efficiency.** (A) Hep2 cells were infected with sh*Non* or sh*Tip30*. DSCs were infected with LV*Non* or LV*Tip30*.TIP30 expression was detected by qRT-PCR after 5 days’ infection. (*n* =3). (B) Hep2-sh*Non* or Hep2-sh*Tip30* cells were transfected with siRNA targeting β-Catenin (si-*CTNNB1-554* or si-*CTNNB1-689*) or scrambled siRNA (si-*NC*). TIP30 and CTNNB1 mRNA levels were detected by qRT-PCR. (*n* =3). (C) DSCs-LV*Non* or DSCs-LV*Tip30* cells were transfected with pcDNA3 or pcCTNNB1.TIP30 and CTNNB1 mRNA levels were detected by qRT-PCR. (*n* =3). ***P* < 0.01

**Supplementary Table**

**Supplementary Table1** The correlation between TIP30 levels and p-AKT or β-catenin expression in LSCC tissue

| Variable | Low  expression of TIP30 (n=46) | High  expression of TIP30 (n=59) | *P* value | *r* |
| --- | --- | --- | --- | --- |
| p-AKT |  |  | **＜0.001** | 0.452 |
| High | 35 | 18 |  |  |
| Low | 11 | 41 |  |  |
| β-catenin |  |  | **＜0.001** | 0.454 |
| Aberrant | 27 | 9 |  |  |
| Normal | 19 | 50 |  |  |

**Supplementary Table 2. Univariate analyses of factors associated with**

recurrence-free survival and overall survival

|  | RFS | |  | OS | |
| --- | --- | --- | --- | --- | --- |
| Variables | Hazard ratio(95% CI) | *P* value |  | Hazard Ratio(95% CI) | *P* value |
| *Gender*  (female vs male) | 1.096(0.428-2.809) | 0.848 |  | 1.195(0.414-3.445) | 0.742 |
| *Age, y*  (＞60 vs ≤60) | 1.099(0.577-2.094) | 0.774 |  | 1.238(0.580-2.645) | 0.581 |
| *T stage*  (T1-T2 vs T3-T4) | 0.610(0.318-1.169) | 0.136 |  | 0.587(0.277-1.241) | 0.163 |
| *Lymph node metastasis*  (N- vs N+) | 0.512(0.271-0.969) | **0.040** |  | 0.484(0.229-1.025) | 0.058 |
| *Clinical stage*  (I-II vs III-IV) | 0.778(0.411-1.470) | 0.439 |  | 0.624(0.292-1.332) | 0.222 |
| *Tumor site*  (Subglottic vs Glottic，Supraglottic) | 2.236(1.177-4.246) | **0.014** |  | 2.688(1.256-5.750) | **0.011** |
| *Histological differentiation*  (G1-G2 vs G3-G4) | 0.166(0.073-0.377) | **0.000** |  | 0.222(0.090-0.547) | **0.001** |
| *TIP30*  (high vs low) | 0.374(0.193-0.723) | **0.003** |  | 0.422(0.198-0.903) | **0.026** |

**Abbreviations:** RFS, recurrence-free survival; OS, overall survival; 95%CI, 95% confidence interval .

Univariate analysis, Cox proportional hazards regression model

**Supplementary Table 3.** **Multivariate analyses of factors associated with recurrence-free survival and overall survival**

|  | Hazard ratio(95% CI) | *P* value |
| --- | --- | --- |
| **RFS** |  |  |
| *Lymph node metastasis*  (N- vs N+) | 0.427(0.224-0.813) | **0.010** |
| *Histological differentiation* |  |  |
| (G1 vs G2) | 0.112(0.048-0.264) | **0.000** |
| *TIP30*  (high vs low) | 0.250(0.125-0.499) | **0.000** |
| **OS** |  |  |
| *Histological differentiation*  (G1-G2 vs G3-G4) | 0.186(0.074-0.463) | **0.000** |
| *TIP30*  (high vs low) | 0.331(0.153-0.715) | **0.005** |

**Abbreviations:** RFS, recurrence-free survival; OS, overall survival; 95%CI, 95% confidence interval

Multivariate analysis, Cox proportional hazards regression model

Variables were adopted for their prognostic significance by univariate analysis and no obvious correlation between each other.

**Supplementary Table 4. Patient characteristics**

| **Characteristics** | **No.of patients(%)** |
| --- | --- |
| *Gender* |  |
| Male | 92(87.6) |
| Female | 13(12.4) |
| *Age, y* |  |
| ≤60 | 46 (43.8) |
| ＞60 | 59(56.2) |
| *T stage* |  |
| T1-T2 | 73(69.5) |
| T3-T4 | 32(30.5) |
| *Lymph node metastasis* |  |
| N- | 62(59.0) |
| N+ | 43(41.0) |
| *Clinical stage* |  |
| I-II | 54(51.4) |
| III-IV | 51(48.6) |
| *Tumor site* |  |
| Subglottic | 41(39.0) |
| Glottic | 50(47.6) |
| Supraglottic | 14(13.3) |
| *Histological differentiation* |  |
| G1-G2 | 52(49.5) |
| G3-G4 | 53(50.5) |
